# A Case of Intraoperative Bladder Rupture During Kidney Transplantation

**DOI:** 10.1002/iju5.70053

**Published:** 2025-05-26

**Authors:** Kosuke Ogawa, Yusuke Tabuchi, Takahiro Yamaguchi, Ryo Iguchi, Hitomi Miyata, Noriyuki Ito, Kazutoshi Okubo

**Affiliations:** ^1^ Department of Urology Kyoto Katsura Hospital Kyoto Japan; ^2^ Department of Nephrology Kyoto Katsura Hospital Kyoto Japan; ^3^ Department of Urology Japanese Red Cross Wakayama Medical Center Wakayama Japan

**Keywords:** anuria, bladder atrophy, bladder rupture, kidney transplantation, ureteroureterostomy

## Abstract

**Introduction:**

Bladder rupture is a rare complication of kidney transplantation, with an increased risk in patients with low‐compliance bladders.

**Case Presentation:**

A man in his 40s with end‐stage renal disease secondary to diabetic nephropathy underwent ABO‐incompatible living‐donor kidney transplantation. He had been anuric for 3.5 years. Bladder rupture was identified during saline injection into the bladder before ureterovesical anastomosis was performed. It was repaired using full‐thickness sutures, and the surgical approach was changed from ureterovesical anastomosis to ureteroureterostomy. The catheter was removed on POD 21, and voiding improved over time without complications.

**Conclusion:**

In patients with bladder atrophy, preventing sudden increases in intravesical pressure and ensuring careful catheter handling are crucial for avoiding bladder rupture.

**Summary**

- Perioperative bladder rupture can occur during kidney transplantation, particularly in patients with bladder atrophy.
- In this case, the rupture likely resulted from a rapid increase in intravesical pressure during saline injection or mechanical injury caused by the urethral catheter.
- The rupture was repaired using full‐thickness sutures, and ureteroureterostomy was performed to avoid direct bladder manipulation.
- Careful catheter handling and controlled saline injections are essential for preventing complications in patients with low‐compliance bladders.

## 1 Case Presentation

The patient was a man in his 40s with end‐stage renal disease caused by diabetic nephropathy. He had been on peritoneal dialysis since June 2018 and was transitioned to hemodialysis in January 2019. His medical history included diabetes, hypertension, and hyperlipidemia, for which he had been receiving pharmacological treatment. Diabetes was diagnosed in 2004, and his HbA1c levels were well‐controlled in the mid‐6% range with a DPP‐4 inhibitor at the time of presentation. He had been anuric for 3.5 years before transplantation. An ABO‐incompatible living‐donor kidney transplantation from his wife (blood type O+ to A+) was planned. Flow cross‐matching testing revealed negative results for both T and B lymphocytes. Tacrolimus and mycophenolate mofetil (MMF) therapy was initiated 5 days before surgery, and steroid therapy was initiated on the day of surgery. The transplantation procedure was performed on January 2024.

## 2 Surgical Techniques

After the induction of general anesthesia, a 16Fr urethral catheter was inserted. The peritoneal dialysis catheter was removed, and a transplantation bed was created in the right iliac fossa. The Lich–Gregoir technique was planned for ureterovesical anastomosis. At our institution, 100–150 mL of saline is slowly injected manually through a catheter tip until bladder distension is confirmed. In this case, although the bladder initially distended after injecting 150 mL of saline, it subsequently collapsed. Additional saline injections failed to restore inflation. Cystoscopy revealed two mucosal tears in the left posterior bladder wall with exposed deep fatty tissue. Retroperitoneal bladder rupture was diagnosed after the exclusion of intraperitoneal fluid accumulation. The rupture site was repaired using full‐thickness, continuous sutures with 2–0 Vicryl under cystoscopic guidance. No leakage was observed at the repair site. Subsequently, urinary reconstruction involved end‐to‐end ureteroureterostomy between the native and graft ureters, which was performed with interrupted sutures using 5–0 PDS, along with the placement of a 5Fr double‐J stent placed to complete the procedure.

## 3 Postoperative Course

**FIGURE 1:**
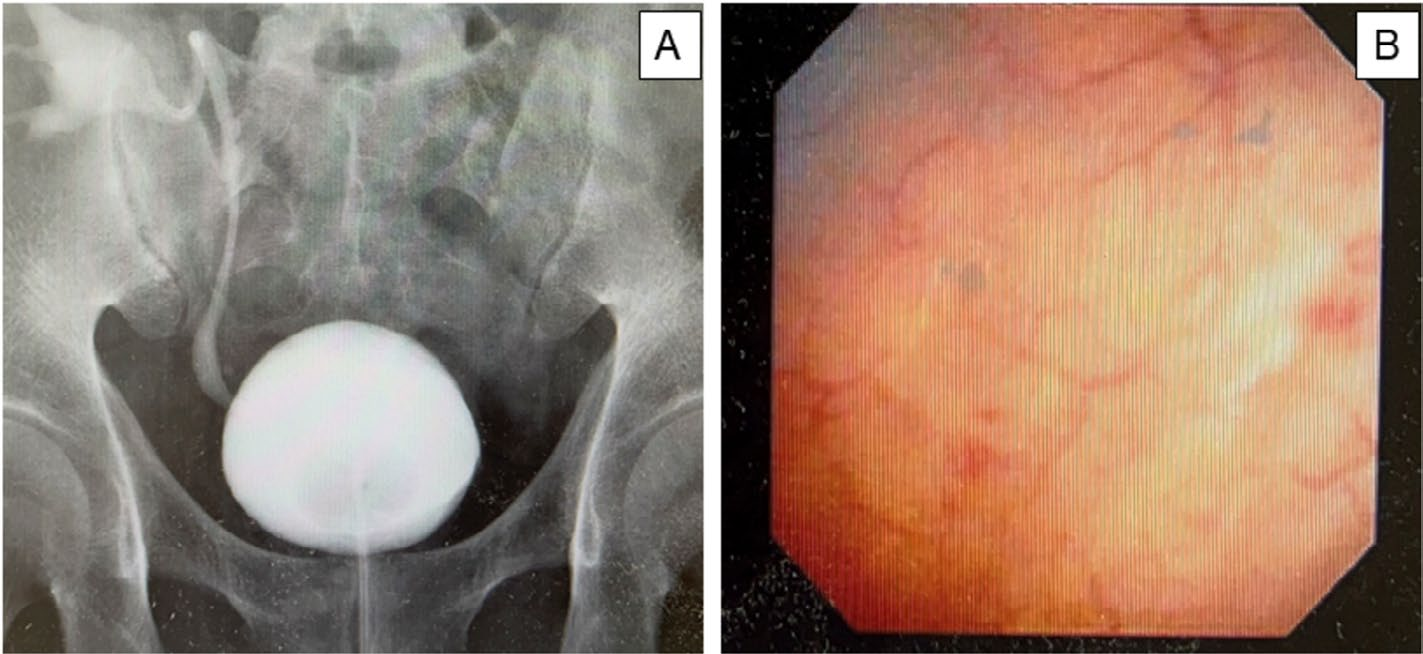
(A) Cystography on POD 13 demonstrates no leakage of contrast media. A double J catheter is indwelled, and vesicoureteral reflux is observed. (B) Cystoscopy on POD 55 confirmed complete healing of the bladder mucosa at the rupture site.

On POD 13, a cystography was performed using a 120 mL saline injection, revealing no leakage (Figure 1A). Oral solifenacin and mirabegron were initiated to lower intravesical pressure and reduce strain on the injury site, and the urethral catheter was removed on POD 21. Subsequently, the initial voided volume was 100 mL with urinary frequency, then increased up to 200 mL, which could be stored with no residual urine by POD 34. The patient was discharged without any recurrence of bladder rupture or urinary complications. On POD 55, the double‐J stent was removed, and a cystoscopy confirmed complete healing of the bladder mucosa at the rupture site (Figure 1B). Mirabegron was given for 4 weeks and solifenacin for 14 weeks to improve bladder compliance and reduce strain on the injury site.

Postoperatively, immunosuppressive therapy was initiated using a combination of tacrolimus, MMF, and prednisolone. Biopsy of the transplanted kidney during follow‐up revealed calcineurin inhibitor nephropathy, leading to the addition of everolimus to reduce the tacrolimus dosage. 3 months postoperatively, maintenance immunosuppression therapy included everolimus (1 mg/day), tacrolimus (3 mg/day), MMF (1500 mg/day), and prednisolone (4 mg/day). 1 year postoperatively, no hydronephrosis was observed in the transplanted kidney.

## 4 Discussion

Bladder rupture during the perioperative period of living kidney transplantation is rare. This is the first report of intraoperative bladder rupture, as previous reports have been limited to postoperative occurrence. Table 1 provides a summary of these cases, including the present case [1, 2, 3]. Brooks et al. and Kim et al. have reported increased intravesical pressure as a contributing factor [1, 2]. Long‐term dialysis patients are known to experience bladder atrophy, resulting in decreased bladder compliance [4]. Patients undergoing dialysis for > 5 years often exhibit significant reductions in bladder capacity and compliance [5]. These cases also involved long‐term dialysis patients with likely bladder atrophy or low compliance. Similarly, our patient, who had a bladder capacity of 100 mL after catheter removal, likely had low compliance.

**TABLE 1:** Case reports of bladder rupture in the perioperative period of kidney transplantation.

Author	Age	Sex	History of dialysis (year)	Onset period	The presumed cause of bladder rupture	Location within the bladder	Intraperitoneal or retroperitoneal	Treatment procedure
Brooks et al. [1]	64	Male	6	POD 16	Increased bladder pressure associated with voiding dysfunction	Dome	Intraperitoneal	Full‐thickness continuous suturing with 3–0 barbed suture under laparoscopy
Kim et al. [2]	26	Male	4	POD 8	Increased bladder pressure after balloon removal or irritation from the Double‐J stent	Left wall	Retroperitoneal	Placement of an indwelling urethral catheter
Jackson et al. [3]	44	Female	2	POD 90	Bladder wall weakening caused by cytomegalovirus infection	Right wall	Retroperitoneal	Full‐thickness suturing with 0‐chromic catgut suture
Our case	40s	Male	6	Intraoperative	Increased bladder pressure due to saline injection during ureterovesical anastomosis or mechanical stimulation caused by the indwelling catheter	Left wall	Retroperitoneal	Full‐thickness suturing with 2–0 Vicryl suture

Abbreviation: POD, postoperative day.

In normal bladders, a rupture threshold of approximately 300 cmH_2_O has been reported [1, 6]. However, in low‐compliance bladders, Blok et al. described a case of bladder rupture after augmentation ileo‐cystoplasty at a detrusor pressure of 52 cmH_2_O. Similarly, Ehdaie et al. reported bladder rupture during filling cystometry in an auto‐augmented bladder at a detrusor pressure of 65 cmH_2_O, with bladder compliance of 6.2 mL/cmH_2_O. These findings suggest that patients with low‐compliance bladders are at a significantly increased risk of rupture even at moderate pressures.

In our case, injecting 150 mL of saline into a bladder with a capacity of only 100 mL may have been excessive. Furthermore, rapid saline injections may sharply increase intravesical pressure, causing bladder wall damage.

To further minimize the risk of iatrogenic bladder injury, pre‐transplant urodynamic studies and cystography can be performed to assess bladder capacity and compliance. These measures can help identify patients at greater risk of bladder complications, allowing for tailored perioperative management strategies, such as reducing the volume of saline instilled during surgery, to reduce injury risk.

In addition to elevated intravesical pressure, mechanical factors may also contribute to bladder rupture. Kim et al. [2] reported that mechanical irritation caused by the double‐J stent could have caused bladder rupture. In our case, catheter insertion might have involved mechanical injury to the bladder wall.

Bladder rupture repair depends on the extent and location of injury. Kim et al. [2] reported a case of retroperitoneal bladder rupture treated conservatively with a urethral catheter, in which leakage resolved by Week 6. Conversely, Brooks et al. [1] reported laparoscopic repair of an intraperitoneal rupture using sutures. Davis et al. reported that a 3 cm bladder rupture required surgical repair, which was complicated by wound dehiscence and necessitated two additional surgeries. The authors attributed the delayed healing to CMV infection, which was potentially exacerbated by steroid‐based immunosuppressive therapy [3]. These cases suggest transplantation‐related bladder ruptures may require longer treatment periods. In our case, the bladder rupture was retroperitoneal but was identified intraoperatively, leading to the decision to perform surgical repair, which was successful.

For urinary tract reconstruction, we opted for ureteroureterostomy using the native ureter. Although the Lich–Gregoir technique is effective in minimizing the risks of leakage and stenosis [7], creating a new anastomosis to the atrophic bladder resulted in a high risk of urinary complications [8]. In our case, both bladder atrophy and intraoperative bladder injury were present, which led us to choose ureteroureterostomy rather than creating a new anastomosis to the bladder. The patient did not develop any urinary complications. Urinary reconstruction using ureteroureterostomy may be a suitable option for bladder rupture repair in patients with low‐compliance bladders.

## 5 Conclusion

Here, we report a rare case of bladder rupture that occurred during living‐donor kidney transplantation. Bladder rupture was likely due to a rapid intravesical pressure increase caused by saline injection or mechanical injury caused by the catheter. In patients with a long history of dialysis, careful handling of the bladder is crucial, and ureteroureterostomy may be considered an alternative option in cases of bladder injury.

## Ethics Statement

The authors have nothing to report.

## Consent

Informed consent was obtained from the patient for the publication of this article and the accompanying images.

## Conflicts of Interest

The authors declare no conflicts of interest.

## References

[1] J. H. Brooks , J. Davies , S. Hishikawa , and J. A. Roberts , “Intraperitoneal Bladder Rupture in the Postrenal Transplant Period,” ACS Case Reviews in Surgery 4, no. 5 (2024): 5–9.

[2] I. Y. Kim , S. B. Lee , B. K. Choi , et al., “Bladder Rupture in Immediate Postrenal Transplant Period of Uncertain Cause,” Experimental and Clinical Transplantation 10 (2012): 180–182. 22432765 10.6002/ect.2011.0089

[3] J. L. Davis and C. O. Callender , “Spontaneous Bladder Rupture and Cytomegalovirus Infection Complicating Renal Transplantation: Cause or Coincidence?,” Journal of the National Medical Association 74 (1982): 1131–1135. 6294313 PMC2561351

[4] K. Hotta , M. Miura , Y. Wada , et al., “Atrophic Bladder in Long‐Term Dialysis Patients Increases the Risk for Urological Complications After Kidney Transplantation,” International Journal of Urology 24 (2017): 314–319. 28190268 10.1111/iju.13297

[5] T. Inoue , N. Imai , S. Kurokawa , et al., “Cystometric Evaluation of Recovery in Hypocompliant Defunctionalized Bladder as a Result of Long‐Term Dialysis After Kidney Transplantation,” Transplantation Proceedings 43 (2011): 1618–1622. 10.1111/iju.13122 27195975

[6] M. M. Agarwal , S. K. Singh , V. Naja , et al., “Spontaneous Bladder Rupture: A Diagnostic and Treatment Dilemma Case Studies and Literature Review,” UroToday. International Journal, ahead of print, 2, no. 2 (2009), 10.3834/uij.1944-5784.2009.04.04.

[7] European Association of Urology (EAU) , EAU Guidelines on Renal Transplantation (EAU Guidelines Office, 2024).

[8] A. Brescacin , S. Iesari , S. Guzzo , et al., “Allograft Vesicoureteral Reflux After Kidney Transplantation,” Medicina 58, no. 1 (2022): 81. 35056389 10.3390/medicina58010081 PMC8780114

